# Characteristics of Child-Rearing Environments Related to Social Development in Early Childhood

**DOI:** 10.3390/children9060877

**Published:** 2022-06-12

**Authors:** Yuko Sawada, Toshiko Katayama

**Affiliations:** 1 Department of Physical Therapy, Morinomiya University of Medical Sciences, 1-26-16, Nanko-kita, Suminoe-ku, Osaka 559-8611, Japan; 2 Higashikohama Kindergarten Affiliation, 2-11-14, Higashikohama, Sumiyoshi-ku, Osaka 558-0051, Japan; toshiko_katayama_room@yahoo.co.jp

**Keywords:** early childhood, social development, childrearing environment

## Abstract

This study aimed to determine the characteristics of childrearing environments related to social development in early childhood. A questionnaire survey was conducted with the caregivers of children attending an urban preschool to identify the characteristics of the childrearing environment in relation to social development in early childhood. The TK Infant Development Test was used to assess social development. The Index of Child Care Environment (13 items in four domains) was used to assess the childrearing environment. Six of the items were used to assess parent–child interaction at home. The correlation coefficients between the social development and childrearing environment items were calculated. Multiple regression analysis was conducted with social development (DQ) as the dependent variable and the childcare environment items as the independent variables. Two types of analyses were conducted: forced entry (model 1) and stepwise (model 2). The results of our univariate and multivariate analyses showed a significant association between the social development items and childrearing environment items after adjusting for the target attributes. This finding suggests that an appropriate childrearing environment promotes social development in early childhood.

## 1. Introduction

Children develop remarkably from infancy to early childhood. The social development nurtured in infancy influences the child’s later development as a person and a member of society, and is especially important during infancy, when children begin to live in groups and interact with their peers [1]. Social development is the result of complex interactions [2,3]. Erikson divided life into eight stages and set developmental tasks to be accomplished at each stage, pointing out the significant social influences on lifelong development. In particular, he stated that the developmental task of infancy, the beginning of life, is basic trust, which refers to the trust relationships that are established with significant others. The “other” here often indicates the caregiver [4]. Thus, infants’ interpersonal relationships begin with their relationships with their caregivers. The emotional bond with the caregiver is called attachment [5], and a stable attachment is established when the caregiver is emotionally responsive [6]. Early childhood is often influenced by the family, with children spending a great deal of time with their caregivers. For this reason, a desirable nurturing environment at home is thought to be associated with the development of social skills. Many findings have been accumulated by large projects abroad. The National Institute of Child Health and Human Development (NICHD) Early Child Care Research Network demonstrated that childcare services [7,8,9,10] and childrearing environments [11,12,13,14,15,16] influence later development.

In these studies, social skills were mentioned, along with intellectual development, as the results of early childhood care and a nurturing environment. Social development is generally defined as the ability to behave in accordance with the lifestyle, value norms, and standards of behavior dictated by the society to which one belongs, and these aspects are easily culturally influenced. In Japan, several studies on social development and environment suggest that a good parenting environment has a positive impact on social development [17,18,19,20]. However, most of the previous studies were conducted with children under the age of three, and no conclusions on the relationship between the childrearing environment and children’s social development have been drawn for studies on infancy in general [21,22,23]. In addition, these were large-scale studies that did not adjust for the quality of childcare, and therefore, the analyses were insufficient to focus attention on family influences. It is necessary to examine the importance of the quality of involvement in a home by considering its influence, while keeping the quality of childcare as constant as possible.

For this reason, the purpose of this study was to clarify the characteristics of the childcare environment in relation to social development in early childhood through a questionnaire survey of caregivers of all children attending the same childcare center with the same childcare environment. The hypothesis of this study is that there is a positive and high association between the influence of home and the development of social skills in a constant childcare environment.

## 2. Materials and Methods

### 2.1. Procedure

A questionnaire survey was conducted at a preschool in Osaka, Japan in January 2021. The survey was administered to caregivers regarding the children’s daily activities. The survey items were age (months), gender, social development, and the childrearing environment. 

### 2.2. Participants

The caregivers of 202 children enrolled in an urban preschool as of January 2021 were provided with the questionnaire survey. The studied preschool is located in a residential area in the suburbs of a large city in Osaka. The preschool has a track record of cooperating in surveys of children’s motor and social development for about 10 years, and both parents and caregivers completely understood the purpose of the study. Although there is no monetary compensation for survey cooperation, there is a commitment to provide parents and caregivers with feedback on the results of developmental testing for those who cooperate in the surveys. Two-hundred completed copies of the survey (response rate = 99%) were received without any missing information on the analysis items. The main respondents were mothers (198, 99%). Consequently, 200 children were included in this analysis (92 boys and 108 girls). The mean age (months) was 60.7 ± 11.8 (range 34–81).

### 2.3. Measures

#### 2.3.1. Social Development

Social development was assessed using the TK Infant Development Test [24]. This test is a multidimensional indicator of infant development through social behavior. It was developed at the Tanaka Institute of Education and has been validated for both reliability and validity. The test targets infants aged 3–7 years and is widely used. This test is an acceptable self-administered test. Familiar caregivers answered the questionnaire in this study. It can be used to understand child development from two aspects: the six domains of “life skills”, which examine the development of social skills and abilities required in daily life, and the five domains of “daily habits”, which assess the acquisition of basic habits and discipline. In this study, the developmental quotient calculated by synthesizing the above items was used as the numerical value of social development.

#### 2.3.2. Home-Rearing Environment

We used the Index of Child Care Environment (ICCE) developed by Anme et al. [17,25,26,27], to evaluate the qualitative and quantitative aspects of a child’s relationship with their environment. This test is an acceptable self-administered test. This scale consists of 13 items belonging to four domains—namely human involvement, avoidance of restriction and punishment, social involvement, and social support—based on the framework of the Home Observation for Measurement of the Environment [28]. Human involvement includes opportunities to play with children and opportunities for spouses to help with childcare. Social involvement includes opportunities to go shopping or to a park with the child. Social support includes the availability of childcare supporters and counselors. 

It must be noted that the survey was conducted in January 2021, at a time when behavioral restrictions had been implemented by the government in response to the COVID-19 pandemic. Therefore, to consider the changes resulting from the behavioral restrictions, the survey items were related to the relationship between caregivers and children at home. The survey included the following six items: “opportunities to play with children”, “opportunities to read books to children”, “opportunities to sing songs with children”, “opportunities for spouses (or substitutes) to help with child care”, “opportunities to have meals as a family” and “opportunities for spouses (or substitutes) to help with child care.” Each item was classified into five levels according to the frequency of involvement as “seldom (1)”, “one to three times a month (2)”, “once or twice a week (3)”, “three or four times a week (4)” and “almost every day (5).”

### 2.4. Ethical Considerations

This study was conducted at an urban preschool, where consent for the survey was obtained. Written and verbal explanations regarding the survey were provided to the caregivers through the preschool, and their written consent was obtained before the study was conducted. To protect their personal information, respondents’ names were anonymized by assigning each one an ID number. Individual envelopes were used for survey collection to prevent the disclosure of respondent identity. The study was approved by the Medical Review Committee of Morinomiya Medical University (Approval No. 2019-073).

### 2.5. Statistical Analysis

The correlation coefficients between the social development and childrearing environment items were calculated. Multiple regression analysis was conducted with social development (DQ) as the dependent variable and the childcare environment items as the independent variables. Two types of analyses were conducted: forced entry (model 1) and stepwise (model 2). In the data analyses, SPSS ver. 27 was used for statistical processing.

## 3. Results

Simple results of the questionnaire survey are shown in Table 1. The relationship between social development and childrearing environments is shown in Table 2. Significant correlations were found for two items. Both were positively correlated, indicating an association between a higher frequency of involvement and a higher developmental quotient. 

The results of the multiple regression analysis regarding the relationship between social development and childrearing environments are presented in Table 3. In the multiple regression analysis, social development was used as the objective variable: the higher the frequency of parent–child interactions, the higher the developmental age. In the forced-injection model (model 1), two items, “age in months” (β = 0.64) and “opportunity to sing songs with the child” (β = 0.20), showed a significant correlation with a contribution rate (R2) of 0.40. In the stepwise model (model 2), three items showed significant associations: “age in months” (β = 0.64), “opportunity to sing songs with children” (β = 0.21), and “opportunity for spouse (or substitute) to help with child care” (β = 0.13), with an R2 of 0.41.

## 4. Discussion

The rapid decline in the birthrate and the shift to nuclear families have led to a growing interest in creating an environment conducive to the healthy upbringing of children. There is a great deal of interest in the factors that critically influence child development, and in recent years, a combination of factors has been examined to study the influence [29]. Humans are social beings who grow and develop immediately after birth, receiving encouragement from those around them [30]. In a number of large-scale studies, childcare and family have been validated as predictors of subsequent growth [7,8,9,10,11,12,13,14,15,16]. The environment in which children are raised and the quality of childrearing are important for their healthy growth. However, due to the nature of these large-scale studies, they involved children and caregivers who belonged to multiple childcare facilities, making it difficult to ensure that the quality of care was consistent. This study provides valuable insights into the relationship between home and social skills while maintaining the same quality of care. This study focused on social development in early childhood. In particular, it focused on the social development of infants by examining the characteristics of the childrearing environment. The analysis results showed that there were significant relationships between several items, indicating the significance of mutual relationships with caregivers in children’s social development. These results were similar to those of previous studies.

Among the different childrearing environment items, caregivers spending time with children and doing things together, such as “opportunities to read books to children” and “opportunities to sing songs with children”, was found to be related to language development, physical development, and lifestyle. The results in previous studies have also shown that interacting and sharing time with caregivers in the home environment, and during play, are positively related to children’s social development [18,19,20,21,22,23].

Furthermore, opportunities for spousal (or substitute) cooperation in childcare, opportunities to have family meals, and opportunities to talk to spouses (or substitutes) about children were shown to be related to many items, such as physical development, language development, group living, and lifestyle. Sugisawa et al. found that spousal participation in childrearing affects social development. Moreover, many other studies have shown that the participation of both the parents in childrearing and an appropriate relationship between the parents and the child affect the latter’s social and cognitive development [31,32,33]. The results of this study suggest that the participation of parents in childrearing has a positive effect on the social development of children, as it allows for varied stimulation and reduces the burden of child rearing [34,35,36,37]. Confirming the results of previous studies, this study also shows that continuous interaction with family is essential for the child’s social development. These results verify the hypothesis of this study that there is a strong relationship between the influence of the home and the development of social skills in a constant childcare environment, and that the provision of an appropriate home environment is related to appropriate upbringing.

The limitations of this study are as follows: first, as this was a cross-sectional study, it did not examine the effects of the childrearing environment. However, since the data of the children associated with the same institution were analyzed and the analysis was conducted by age, the authors believe that this study was able to examine the effect of age on social development. Furthermore, in longitudinal studies, it is sometimes difficult to obtain continuous cooperation and the number of research subjects can be small. However, in this study, the response rate was high at 99%, and the authors believe that they were able to grasp the trend of the entire preschool. Second, there was insufficient information on the target attributes. The survey was designed to minimize the number of items to ensure the ease of collection, which is an important factor. However, since this study examined the relationship between the childrearing environment at home and social development, a greater amount of knowledge could have been obtained by analyzing the detailed birth order, family composition, economic status, and social information of the caregivers. In the future, the authors plan to obtain the permission of the preschools and caregivers, add additional information, and conduct a reanalysis for further detailed investigation. Third, COVID-19 impacted how this survey was conducted. The survey was conducted in January 2021, when children’s activities were limited to indoors to prevent the spread of the virus. Originally, for the evaluation of the childcare environment, the survey included many items such as the frequency of going out and interactions with other people, as previous studies had pointed out the relationship between these items and a child’s development. However, the behavioral restrictions greatly limited children’s frequency of going out and interactions with other individuals. Therefore, these items were excluded from the survey. Therefore, the authors believe that the impact of the spread of coronavirus infection was minimized. Fourth, this survey was self-administered by parents. Since a nonprofessional evaluator was required to evaluate their own child, the test results may not be consistent. However, both the TK and ICCE are self-administered tests, and their reliability and validity have been verified. The reliability of the results is maintained. Instead, information was collected on interactions at home and found that they were positively related to children’s development, indicating that interactions between caregivers and children at home promote the latter’s social development. Fifth, the subject population of this study is small. This is a result of the importance of keeping childcare constant, a characteristic of the study. For this reason, statistical analysis was not adequate. However, by including detailed tables of responses and describing the results of individual tests, the study attempted to accurately present the subject characteristics. In addition, the data were obtained while keeping childcare constant, which was the purpose of the study. Data accumulation and devising statistical methods are issues to be addressed in ongoing research.

## 5. Conclusions

Our study analyzed the relationship between social development in early childhood and childrearing environments by conducting a survey with 200 preschool-attending children aged 3–5 years. The univariate and multivariate analyses in this study showed that there was a significant correlation between the social development items and the childrearing environment, adjusting for the target attributes. This finding indicates that an appropriate childrearing environment has a positive impact on social development in early childhood.

## Figures and Tables

**Table 1:** Simple results of the questionnaire survey.

	Mean	SD	Min.	Max.
Social development				
Developmental Quotient	91	22.6	44	155
Childrearing environment				
Play with your child	4.41	0.851	2	5
Read books to your child	3.02	1.317	1	5
Sing songs with your child	3.97	1.190	1	5
Work together with your partner to raise	3.90	1.299	1	5
Eat meals together as a family	4.42	0.953	1	5
Talk with your partner about your child	4.14	1.150	1	5

**Table 2:** The relationship between social development and childrearing environment.

	Correlation Coefficient	*p* Value
Play with your child	0.120	0.090
Read books to your child	0.075	0.290
Sing songs with your child	0.212	0.003
Work together with your partner to raise	0.148	0.037
Eat meals together as a family	0.077	0.280
Talk with your partner about your child	0.101	0.156

**Table 3:** Association between social development and parenting environment (multiple-linear regression).

	Model 1		Model 2	
	Standardization Factor (β)	*p* Value	Standardization Factor (β)	*p* Value
Constant		0.58		0.12
Age (months)	0.64	0.00	0.64	0.00
Play with your child	0.03	0.64		
Read books to your child	0.04	0.46		
Sing songs with your child	0.20	0.00	0.21	0.00
Work together with your partner to raise	0.11	0.07	0.13	0.02
Eat meals together as a family	0.04	0.54		
Talk with your partner about your child	−0.01	0.92		
Adjusted R2 multiplier	0.40		0.41	
Significance probability	0.0001		0.0001	

## Data Availability

Not applicable.
